# Mucopolysaccharidosis IIIB, a lysosomal storage disease, triggers a pathogenic CNS autoimmune response

**DOI:** 10.1186/1742-2094-7-39

**Published:** 2010-07-16

**Authors:** Smruti Killedar, Julianne DiRosario, Erin Divers, Phillip G Popovich, Douglas M McCarty, Haiyan Fu

**Affiliations:** 1 Center for Gene Therapy, The Research Institute at Nationwide Children's Hospital, Columbus, Ohio, USA; 2 Department of Pediatrics, College of Medicine, the Ohio State University, Columbus, Ohio, USA; 3 Department of Molecular Virology, Immunology & Medical Genetics, College of Medicine, the Ohio State University, Columbus, Ohio, USA; 4 Department of Neuroscience, College of Medicine, the Ohio State University, Columbus, Ohio, USA; 5 Center for Brain and Spinal Cord Repair, College of Medicine, the Ohio State University, Columbus, Ohio, USA

## Abstract

**Background:**

Recently, using a mouse model of mucopolysaccharidosis (MPS) IIIB, a lysosomal storage disease with severe neurological deterioration, we showed that MPS IIIB neuropathology is accompanied by a robust neuroinflammatory response of unknown consequence. This study was to assess whether MPS IIIB lymphocytes are pathogenic.

**Methods:**

Lymphocytes from MPS IIIB mice were adoptively transferred to naïve wild-type mice. The recipient animals were then evaluated for signs of disease and inflammation in the central nervous system.

**Results:**

Our results show for the first time, that lymphocytes isolated from MPS IIIB mice caused a mild paralytic disease when they were injected systemically into naïve wild-type mice. This disease is characterized by mild tail and lower trunk weakness with delayed weight gain. The MPS IIIB lymphocytes also trigger neuroinflammation within the CNS of recipient mice characterized by an increase in transcripts of IL2, IL4, IL5, IL17, TNFα, IFNα and Ifi30, and intraparenchymal lymphocyte infiltration.

**Conclusions:**

Our data suggest that an autoimmune response directed at CNS components contributes to MPS IIIB neuropathology independent of lysosomal storage pathology. Adoptive transfer of purified T-cells will be needed in future studies to identify specific effector T-cells in MPS IIIB neuroimmune pathogenesis.

## Background

Mucopolysaccharidosis IIIB is a lysosomal storage disease (LSD) with severe neurological manifestations[1]. No treatment for MPS IIIB is currently available. The disease is caused by autosomal recessive defects in α-*N*-acetylglucosaminidase (NaGlu), a lysosomal enzyme essential in the stepwise degradation of heparan sulfate (HS), a biologically important glycosaminoglycan (GAG)[1].

The primary pathology of MPS IIIB is the accumulation of HS or HS-derived materials in lysosomes in somatic and central nervous system (CNS)[1,2]. The detailed mechanisms of MPS IIIB pathology, especially neuropathology, remain unclear although recent studies have implicated aberrant activation of innate and adaptive immunity[2-9]. Indeed, immunosupression with prednisolone was shown to delay the progression of the CNS disease in MPS IIIB mice[8], supporting the critical contribution of neuroimmunity to the neuropathology of MPS IIIB.

Although it is unclear how lysosomal HS storage initiates aberrant CNS immune responses, it is perhaps not surprising considering that abnormal GAG metabolism has been described in several autoimmune diseases, including rheumatoid arthritis (RA), scleroderma and systemic lupus erythematosus (SLE)[10-14]. Heparan sulfates are important functional components of numerous glycoproteins/proteoglycans on cell surfaces and in extracellular matrices where they engage in biologically important protein interactions in development and normal physiology. Numerous glycobiology studies have demonstrated the active participation of GAGs and their proteoglycan backbones in leukocyte functions, including antigen presentation and leukocyte homing/transmigration to sites of inflammation[10-12,15-18]. Changes in cell surface HS has been shown to modulate the sensitivity of T-cell responses to diverse activation stimuli[19].

Our recent novel data suggested that enhanced CNS antigen presentation and lymphocyte activation in the brains of MPSIIIB mice may be a critical contributor to the progression of MPS IIIB[8]. Also, the *de novo *synthesis of CNS-specific antibodies in MPS IIIB mice suggests that neuroantigens are probable targets of autoimmune attack during progression of MPS IIIB. A recent study also showed the potential involvement of cytotoxic T-cells in neuropathology of MPS IIIB[9]. To test this hypothesis and thereby confirm lymphocytes as effectors of pathology in MPS IIIB, we isolated lymphocytes from MPS IIIB mice and age-matched wild-type (wt) control mice and then passively immunized naïve wt recipient mice with these activated cells. Herein we present anatomical, molecular and behavioral evidence that the pathological sequelae of MPS IIIB is due in part to the induction of a CNS autoimmune response.

## Methods

### Animals

MPS IIIB knock-out mice [2] were maintained on an inbred background (C57BL/6) by backcrosses of heterozygotes. This colony has been through 16 backcrosses, alternated by a cross with wt C57BL/6 to generate heterozygotes for next backcross. MPS IIIB mice were housed in the barrier area in the Vivarium at the Research Institute at Nationwide Children's Hospital (NCH-RI). Progeny mice were genotyped by PCR. The MPS IIIB mice and their age-matched wt littermates were used in the experiments. All procedures using MPS IIIB mice in this study have been approved by the Institutional Animal Care and Use Committee at NCH-RI. All care and procedures were in accordance with the *Guide for the Care and Use of Laboratory Animals *[DHHS Publication No. (NIH) 85-23]. MPS IIIB mice and control wt littermate were used in all experiments in this study.

### Lymphocyte preparation

Mice were anesthetized with an intraperitoneal injection of Avertin (0.3 - 0.4 μg/g) and spleens were dissected for lymphocyte preparation, following established procedures. 1) Under sterile conditions, cell suspensions in RPMI media containing 2% FBS were filtered through a 70 μm nylon cell strainer. 2) The cells were washed and resuspended in Fac's buffer (PBS, 2% fetal calf serum), 3) then incubated with RBC lysis buffer (eBioscience) to remove red blood cells, and 4) washed and resuspended in Fac's buffer or RPMI media for further analysis. The number of splenocytes was determined using Sysmex KX-2IN Automated Hematology Analyzer after step 1) and again at the end of the process.

### Adoptive transfer

Splenocytes from 2-3-month-old MPS IIIB mice were isolated as described above. Single cell suspension of splenocytes isolated from multiple mice were pooled and stimulated in vitro for 48 hours with Concanavalin A (ConA, 1 μg/ml) in RPMI 1640 growth media (10% FBS, 2 mM L-glutamine, 100 U/ml penicillin 100 μg/ml streptomycin and 50 μM 2-ME). The non-adherent cells were collected and washed with PBS for adoptive transfer. The isolated lymphocytes (2 × 10^7^/mouse) were injected intravenously (IV) into 10-12-week-old wt male littermates through tail vein. The mice were then observed daily for their wellbeing and clinical disease signs over 2-6 weeks. Lymphocytes isolated from age-matched wt littermates were IV injected into age-matched wt littermates as controls.

### Clinical evaluation

Mice were examined daily to identify any abnormalities. After observing a mild ascending paralytic clinical disease in animals receiving MPS IIIB lymphocytes, we adopted a scoring system used in evaluation of experimental autoimmune encephalomyelitis (EAE)[20] to assess mice in this study. The scoring system was on a scale of 0-5: 0 = no clinical disease; 1 = tail weakness; 1.5 = tail paralysis; 2 = hind limb weakness; 3 = hind limb paralysis; 3.5 forelimb weakness; 4 = forelimb paralysis; 5 = moribund or death.

### Grip tests

Grip tests were performed at 14 days after the adoptive transfer to evaluate the strength of limbs, using a digital grip strength meter (Columbus instruments). The grip strength was presented as peak tension (g).

### Activity measures

Exploratory activity in a novel environment was assessed in a one-hour trial in a photobeam open field chamber [21](San Diego instruments). Counts were taken of the number of photobeams broken during the trial, with separate measures for locomotor, vertical, and rearing activity.

### Rotarod

Subjects were tested on an accelerating rotarod (Med Associates Inc.) to assess motor coordination [21]. For the first test session, animals were given three trials, with 45 seconds between each trial. Two additional trials were given 48 hours later. Revolutions per minute (rpm) was set at an initial value of 3, increasing to a maximum of 30 rpm across five minutes (the trial length). Measures were taken for latency to fall from the rotating barrel (sec).

### Tissue sample collection

Tissue samples were collected at 14 days, and 5 weeks after adoptive transfer. The animals were anesthetized with an intrperitoneal injection of 2.5% Avertin, blood samples were collected. The mice were then perfused transcardially with 20 ml of cold PBS (0.1 M, pH7.4), and, for histological examinations, followed by 4% paraformalde, prior to tissue collection. Brain, spinal cord and spleen samples were collected. The half brain samples and spinal cords were stored at -80°C before being processed for total RNA extraction. Tissue samples from paraformaldehyde-perfused animals were further fixed in 4% paraformaldehyde overnight, then infiltrated in 3% sucrose, and then embedded in OCT for cryosectioning, and histology and immunohistochemistry staining. Each assay was performed using samples from at least 4 mice (n ≥ 4/group).

### Flow cytometry (FC)

Flow cytometry was performed on donor splenocytes (4 × 10^6 ^lymphocytes per sample), expanded with ConA, using antibodies against mouse CD3e, CD4, CD8, CD25, CD45R/B220, and F4/80 (BD Biosciences). The labeled cells were sorted using a BD-LSRII flow cytometer (BD Biosciences) and data were analyzed using BD FACSDiva software.

### T-cell proliferation assay

Freshly isolated splenocytes were assayed for T-cell proliferation following published procedures [22,23] using freshly isolated cells with anti-mouse-CD3e antibody (eBioscience) as stimulator, and 3-(4,5-dimethylthiazol-2-yl)-2,5-diphenyltetrazolium bromide (MTT) as cell proliferation marker. Cells in RPMI growth media (2 × 10^5 ^in 200 μl/well, in triplicates or more) were plated onto 96-well tissue culture plates pre-coated with anti-CD3e antibody (50 μl, 10 μg/ml in PBS) or PBS, and incubated at 37°C for 72 hr. The cell clusters formed in each well were then counted under a microscope, before the addition of 20 μl MTT buffer (5 mg/ml in PBS), followed by 4-hour incubation. The MTT lysis buffer (20% SDS in 50% DMF) was then added to each well and incubated at 37°C overnight. T-cell proliferation was expressed as: Stimulation index = A_570 _with anti-CD3/A_570 _with PBS.

### Bio-plex cytokine assay

Splenocytes (10^6 ^cells/ml) were incubated in RPMI 1640 growth media, as described above, in the presence or absence of ConA. Media were collected after 42 hour incubation, and assayed for cytokine production by Bio-plex cytokine assay (BioRad), following the procedures provided by the manufacturer. The Bio-plex panel used targets IL2, IL4, IL5, IL10, IL17, IFNγ and TNFα.

### RNA and qRT-PCR

Total RNA was isolated from half brain of each individual mouse (n = 4/group), using SV Total RNA Isolation System (Promega), following the manufacturer's protocols. The isolated total RNA samples were then further purified through an RNeasy spin column (Qiagen). The mouse brain total RNA was assayed for the expression of immune-associated genes, using sequence specific primers. A pair of primers for murine GAPDH (glyceraldehyde-3-phosphate dehydrogenase) gene was used as internal control. Superscript™ First-strand Synthesis System for RT-PCR (Invitrogen) was used for cDNA synthesis. The qRT-PCR was performed using SYBR^® ^Green PCR Master Mix (Applied Biosystems). Comparative threshold (C_T_) method was used for data analysis. Data were expressed as relative quantitation of gene expression (2^^-ΔΔCt^)[24], in the brain of mice given an adoptive transfer of lymphocytes from MPS IIIB mice vs. lymphocytes from wt mice.

### Immunohistochemistry (IHC)

Mouse brain tissues were processed for serial transverse cryostat sections (10-15 μm). IHC staining was performed following the procedures provided by manufacturers, using primary antibodies against mouse CD3 (AbD Serotec) and CD8 (Biolegend), and secondary antibodies conjugated with horse radish peroxidase or Alexa Fluo 568.

### Statistical analysis

Data are reported as means ± SD. *P*-values between datasets were determined by two-tailed Student's *t-test*. Significance was defined as *P *< 0.05.

## Results

### MPS IIIB causes spontaneous activation of the adaptive immune system

**Figure 1:**
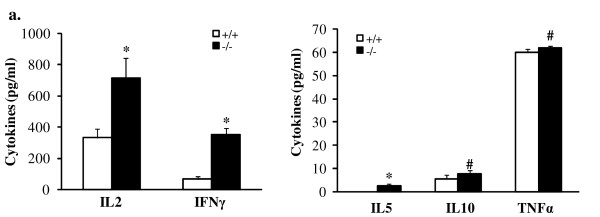
**Enhanced cytokine production by MPS IIIB lymphocytes**. Donor splenocytes isolated from 2-3-month-old MPS IIIB (n = 8) or wt mice (n = 8) were pooled and incubated for 48 hrs in RPMI 1640 growth media in the presence of ConA (1 μg/ml). The media were then assayed for cytokines by Bio-plex assay. The data here were means from two sets of independent experiments (n = 8/group/experiment). Each sample was analyzed in duplicates. **+/+**: wt; **-/-**: MPS IIIB. *: P < 0.01; **#**: P < 0.05.

To test our hypothesis that MPS IIIB primes an autoimmune response which subsequently provokes the onset or exacerbates MPS IIIB neuropathology, we analyzed the phenotype and functional potential of MPS IIIB mouse lymphocytes. T-cell proliferation assay showed a significant increase in stimulation index of splenic lymphocytes from MPS IIIB mice, compared to wt cells, when stimulated with anti-CD3e ex vivo (data not shown), suggesting T-cell activation in MPS IIIB mice. To be used in the adoptive transfer experiments below, splenic lymphocytes isolated from wt (10-12 weeks old) or MPS IIIB mice (n = 8/group) were pooled within a group. Leukocytes from wt or MPS IIIB mice were comprised of a mixed population of T cells, B cells and macrophages (Table 1). Interestingly, the splenic T:B cell ratio was reduced 26% in MPS IIIB mice and was accompanied by an increase in F4/80+ macrophages, suggesting the activation of B-cells and macrophages. T cells from MPS IIIB mice spontaneously (i.e., without *ex vivo *stimulation) secreted more cytokine at higher concentrations than T cells from wt mice. Specifically, we found IL2, IL4, IL5, IL10, IL17 and IFNγ to be present in supernatants of T cells from MPS IIIB mice but not wt mice (data not shown). After stimulation with concanavilin A (ConA), MPS IIIB lymphocytes secreted greater amounts of IL2, IL5, IL10, IFNγ and TNFα as compared to ConA-activated wt lymphocytes (Fig. 1). These data suggest that T cells are activated *in vivo *during the progression of MPS IIIB and develop a predominantly Th1 cytokine profile (i.e., high levels of IL-2 and IFNγ).

**Table 1:** Donor splenic lymphocyte population *Splenocyte population (%)

Donor	**B220**^**+**^	**CD3**^**+**^		**F4/80**^**+**^
			
mice		**CD4**^**+**^	**CD8**^**+**^	
**wt**	47.7	22.2	15.2	8.25
**MPS IIIB**	55.5	14.6	14.5	14.1

*Splenic lymphocytes isolated from age-matched (10-12-week-old) male MPS IIIB or wt mice were pooled and stimulated with ConA (1 μg/ml in RPMI growth media) for 48 hrs. Data represent the average of two independent experiments (n = 8 donors/group/experiment).

### MPS IIIB lymphocytes cause neurological impairment

Adoptive transfer of isolated lymphocytes was performed to assess the pathogenic potential of MPS IIIB lymphocytes. Because our previous studies showed that the CNS immune activation in MPS IIIB mice involves virtually every aspect of the immune system, total mouse splenic lymphocytes were used for adoptive transfer in this study.

**Figure 2:**
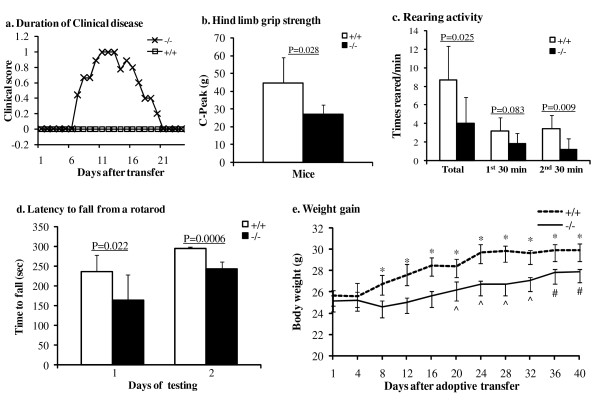
**Pathogenic effects of the adoptive transfer of MPS IIIB lymphocytes**. Splenocytes isolated from multiple 2-3-month-old MPS IIIB mice were pooled and stimulated in vitro with 1 μg/ml Con A for 48 hours. The activated non-adhesive cells were then isolated and injected IV into 8-10-week-old wt mice (n = 20). The controls were the wt mice treated with an IV injection of wt lymphocytes (n = 20). The mice receiving MPS IIIB lymphocytes developed mild EAE-like clinical disease (**a**), with decreased hindlimb grip strength (**b**), impaired rearing activity (**c**), reduced latency on rotarod (**d**), and delayed growth (**e**). **+/+**: adoptive transfer of wt lymphocytes; **-/-**: adoptive transfer of MPS IIIB lymphocytes. *: P < 0.01 wt cell-treated vs. MPS IIIB cell-treated mice;: P < 0.05 vs. day 8; **#**: P < 0.01 vs. day 8. The bw differences of MPS IIIB cell recipients vs. wt cell recipients from day 8-40 were significant (P < 0.05).

Lymphocytes that were pooled from the spleen of age-matched donor MPS IIIB or wt mice were activated with ConA *ex vivo *for 48 hours then were injected intravenously into naïve wt recipient mice (n = 26-38 recipient mice/group, 8-10 weeks old). All recipient mice were monitored daily for signs of neurological impairment using a 0-5 "clinical scale" that is widely used to quantify the magnitude of ascending paralysis in experimental autoimmune encephalomyelitis (EAE)[20]. None of the mice treated with wt lymphocytes developed disease. In contrast, all mice receiving lymphocytes from MPS IIIB donor mice developed mild ascending paralysis characterized by weak tail and lower trunk (score of ~1) (Table 2, Fig. 2a, Additional files 1, 2, 3, 4). Tail weakness emerged 6-9 days after adoptive transfer and persisted for up to 15 days reaching peak disease at ~2 weeks post transfer (Table 2; Fig. 2a).

**Table 2:** Clinical disease induced in naïve wild-type recipient mice by adoptive transfer of lymphocytes from MPS IIIB mice

*Lymphocyte Donor	Disease Incidence at peak (14d pi)	Day of onset	**Mean peak clinical score
+/+	0/20 (0%)	n/a	0
-/-	30/30 (100%)	7.4 ± 1.1	0.9 ± 0.2

In total, 26 and 38 mice received adoptive transfer of wt and MPS IIIB lymphocytes, respectively. Data here include only mice that went through complete disease observation. The rest of mice were terminated for tissue analysis at 1 week post transfer and were not included in this table.

* +/+: wt mice; -/-: MPS IIIB mice. ** Peak: 2 weeks post transfer.

Given the subjective nature of the clinical scale, additional behavioral assays were completed to confirm and quantify the magnitude of neurological impairment. Specifically, we quantified grip strength, volitional open field activity and latency on rotarod at 2 weeks post transfer, i.e., the time of peak clinical disease. As shown in Fig. 2b, hind limb grip strength was significantly impaired in mice injected with MPS IIIB lymphocytes (P < 0.05 vs. mice injected with wild-type lymphocytes). Fore limb grip strength was unaffected (not shown). Hind limb deficits were confirmed in the open field and on the rotorod, where a significant decrease in rearing activity (P < 0.05) and shorter latencies to fall were noted in mice treated with MPS IIIB lymphocytes (Fig. 2c&2d).

To further assess the pathological impact of adoptively-transferred MPS IIIB lymphocytes, we monitored the body weight of mice born on the same day that received either wt or MPS IIIB lymphocytes (n = 6-8 recipient mice/group). All mice had equal access to food and water each day during the analysis period. Mice treated with wild-type lymphocytes increased their body weight ~16% over baseline in 20 days (Fig. 2e). In contrast, mice that received MPS IIIB lymphocytes increased their body weight 5% by the end of the observation period (i.e., 40 days post-transfer). This significant delay in weight gain paralleled the onset and progression of neurological impairment. Collectively, these data suggest that MPS IIIB lymphocytes cause neurological impairment and a sustained sickness response.

### Adoptively transferred MPS IIIB lymphocytes alter CNS gene expression and promote intraparenchymal inflammation and glial activation

**Figure 3:**
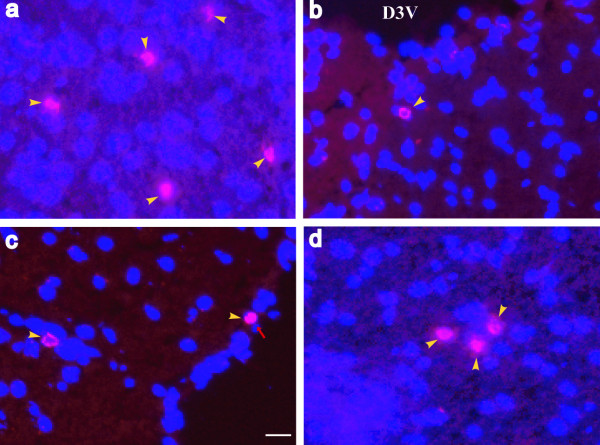
**CD8+ T-cell infiltration in the CNS of mice receiving an adoptive transfer of MPS IIIB lymphocytes**. Cryostat sections of brain and spinal cord of MPS IIIB lymphocyte treated mice (2 wk pi) were assayed by immunofluorescence, using a primary antibody against mouse CD8a and a secondary antibody conjugated with Alexa Fluo 568. a. thalamus; b. brain stem; c. cerebral cortex; d. lumbar spinal cord. Yellow arrowheads: CD8+ T-cells; red arrow: meninge; D3V: dorsal 3rd ventricle. scale bar: 20 μm.

To evaluate the impact of adoptively transferred MPS IIIB lymphocytes on the nervous system of recipient mice, we used qRT-PCR to compare mRNA profiles of brains prepared from mice receiving wild-type or MPS IIIB lymphocytes at the peak of clinical disease (2 weeks post-transfer). We observed a significant up-regulation of markers of innate immunity and glial activation (i.e., CD45, CD68, GFAP) along with a significant increase in expression of multiple cytokines, including IFNγ, IL2, IL4, IL5, IL17, TNFα, IFNα and Ifi30 (Table 3). Western blotting for GFAP confirmed the astrocyte activation (data not shown). We also noted a modest increase in CD3e, CD4 and CD8a mRNA suggesting the presence of T-cell infiltrates in the CNS of mice receiving MPS IIIB lymphocytes (Table 3). Immunofluorescence staining of brain cryosections from recipient mice confirmed this hypothesis showing the presence of CD8+ T-cells in the brain and spinal cord of mice receiving MPS IIIB lymphocytes at 2 weeks (Fig. 3) but not at 4 weeks post transfer. The distribution of these T-cells was sporadic without evidence of profound clustering (as in EAE). Immunoflurescence for CD3 and CD4 was not successful due to the quality of the antibodies that did not even stain cells in spleen. We did not find T cells in the brain or spinal cord of mice receiving injections of wild-type lymphocytes.

**Table 3:** Effect of adoptive transfer of MPS IIIB lymphocytes on the expression of immune-associated genes in the brain of recipient mice

Gene symbol	Gene name	GenBank access #	^**#**^**2^-ΔΔCt ^(fold of increase)**
IFN-γ	Interferon gamma	NM_008337	17.8*
CD68	CD68 antigen	NM_009853	5.2*
GFAP	Glial fibrillary acidic protein	NM_010277	4.1*
IL4	Interleukin 4	NM_021283	2.8*
IL5	Interleukin 5	NM_010558	1.7*
IL6	Interleukin 6	NM_031168	1.3
IL17	Interleukin 17	NM_010552	2.1*
IL2	Interleukin 2	NM_008366	1.9*
TNF-α	Tumor necrosis factor alpha	NM_013693	1.9*
CD45	Protein tyrosine phosphatase receptor type C	NM_011210	1.7*
Ifi30	Interferon gamma inducible protein 30	NM_023065	1.8*
IFN-α	Interferon alpha	NM_008334	1.6*
CD3e	CD3 antigen, epsilon polypeptide	NM_007648	1.3*
CD4	CD4 antigen	NM_013488	1.4*
CD8a	CD8 antigen, alpha chain	NM_001081110	1.2
IL10	Interleukin 10	NM_010548	1.0

^**#**^Real-time qRT-PCR data were analyzed by means of the comparative threshold (CT) method using mouse GAPDH as internal control and expressed as 2^-ΔΔCt^. These changes reflect relative increases in gene expression in the brain of mice receiving MPS IIIB lymphocytes (n = 4) as compared to wild-type lymphocytes-treated mice (n = 4). *P < 0.05 (ΔΔCt of MPS IIIB lymphocytes-treated mouse brains vs. wild-type lymphocytes-treated mouse brains).

## Discussion

Our recent studies have shown that lymphocytosis is a natural consequence of MPS IIIB[8]. In this study, we show that T-cells in MPS IIIB mice become activated spontaneously *in vivo*. Although we have not yet defined the mechanisms responsible for T cell activation in this model, we show for the first time that these cells have the ability to cause neurological impairment when they are adoptively transferred into naïve wt recipient mice. These data suggest that MPS IIIB primes an autoimmune response, which subsequently provokes the onset or exacerbates MPS IIIB neuropathology. Given the important role of HS in modulating the sensitivity of T cells and antigen presenting cells to activation[10-12,19,25-27] and the involvement of abnormal GAG metabolism in autoimmune diseases[10-14], it is likely that lysosomal HS storage initiates aberrant immune responses culminating in the onset of an autoimmune reaction.

Based on our past and present data, we predict that a subset of the activated MPS IIIB lymphocytes are CNS-reactive and that these cells can impair neurological function independent of any neuropathology caused by aberrant lysosomal HS storage. The temporary and mild neurological impairment that we observed in naive wt recipient mice is similar to the course of disease that is induced after transfer of autoimmune lymphocytes from rats with traumatic spinal cord injury[28]. Accordingly, like in that study, it is doubtful that transferred lymphocytes cause demyelination or neuronal injury. Instead MPS IIIB lymphocytes alter brain homeostasis with evidence of glial activation and significantly increased expression of cytokines (IL2, IL4, IL5, IL17, IFNγ) that are known cause pathological changes in experimental models of Th1-mediated CNS autoimmunity[29,30]. Importantly, pro-inflammatory Th1 cytokines can block neuronal function without triggering cell death or overt neuropathology[31]. It is likely that Th17 may also be involved in the adoptive-transfer-mediated change in the CNS, given that IL17-producing CD4^+ ^T-cells have been shown to play a fundamental role in different models of autoimmune diseases[32].

It is worth noting that even though adoptive transfer of MPS IIIB lymphocytes causes global changes in CNS cytokine expression with neurological impairement, these changes are mild compared to those elicited by passive immunization with myelin-reactive T cells and the onset of EAE. Without adjuvant priming - a necessary feature of EAE induction[33-35], the threshold and frequency of CNS-reactive T cells is expected to be lower in MPS IIIB mice. Also, even though it is clear that the induction of an autoimmune response directed at the CNS is associated with aberrant lysosomal HS storage[8], the identities of the autoantigens that trigger this response are currently unknown. For this reason, we were unable to activate MPS IIIB splenocytes ex vivo using antigen-specific stimulation. Instead, ConA was used as a polyclonal stimulant. Thus, only a fraction of transferred lymphocytes would be expected to be pathogenic or specific for CNS proteins, the remaining cells may have regulatory or anti-inflammatory functions as described in other models of nerve injury and autoimmune diseases[36-38].

## Conclusion

We have demonstrated for the first time the pathogenic property of MPS IIIB lymphocytes, a subset of which can traffic to the CNS where they promote neuroinflammation with neurological impairment. The neuropathogenesis of lymphocytes is likely a critical contributor to the neuropathology of MPS IIIB, which is independent of the lysosomal storage pathology. Since the onset of this pathological neuro-immune reaction arises predictably in MPS IIIB mice without concomitant infection or injury, this model may be useful for studying novel signaling pathways and activation mechanisms that are responsible for controlling pathological CNS-immune interactions. Given the central role of T-cells in autoimmune responses, further studies using purified T-cells will be critical for investigating the neuropathogenic role of specific autoimmune effector T-cells in MPS IIIB.

## Competing interests

The authors declare that they have no competing interests.

## Authors' contributions

SK carried out lymphocyte preparation, T-cell proliferation, flow cytometry, Bioplex cytokine assays, and disease evaluation, and participated in data analyses and manuscript preparation. DJ performed behavior testing and qRT-PCR assay. ED participated in lymphocyte preparation and daily observation on subject animals. PP participated in the design of the study, data analyses and manuscript preparation. DM participated in the design of the study and manuscript preparation. HF conceived of the study, participated in its design, performed the adoptive transfer, and took part in data analyses and manuscript preparation. All authors read and approved the final manuscript.

## Supplementary Material

Additional file 1 **Reduced tail strength of mice receiving an adoptive transfer of MPS IIIB mouse lymphocytes**. Splenic lymphocytes (2 × 10^7^) from MPS IIIB mice were injected IV into wt littermates. Video of a MPS IIIB lymphocyte recipient mouse was taken at 2 wk post transfer, showing the decrease in tail strength, compared with the mice treated with wt lymphocytes (Additional files 3 &4). Click here for file

Additional file 2 **Reduced tail strength of mice receiving an adoptive transfer of MPS IIIB mouse lymphocytes**. Splenic lymphocytes (2 × 10^7^) from MPS IIIB mice were injected IV into wt littermates. Video of a MPS IIIB lymphocyte recipient mouse was taken at 2 wk post transfer, showing the decrease in tail strength, compared with the mice treated with wt lymphocytes (Additional files 3 &4). Click here for file

Additional file 3 **Tail strength of mice receiving an adoptive transfer of wt mouse lymphocytes**. Splenic lymphocytes (2 × 10^7^) from wt mice were injected IV into wt littermates. Video of a wt lymphocyte recipient mouse was taken at 2 wk post transfer, showing the tail strength, as a control of MPS IIIB lymphocyte recipient mice (Additional files 1 &2). Click here for file

Additional file 4 **Tail strength of mice receiving an adoptive transfer of wt mouse lymphocytes**. Splenic lymphocytes (2 × 10^7^) from wt mice were injected IV into wt littermates. Video of a wt lymphocyte recipient mouse was taken at 2 wk post transfer, showing the tail strength, as a control of MPS IIIB lymphocyte recipient mice (Additional files 1 &2). Click here for file
